# Mitochondrial phylogenomics and mitogenome organization in the parasitoid wasp family Braconidae (Hymenoptera: Ichneumonoidea)

**DOI:** 10.1186/s12862-022-01983-1

**Published:** 2022-04-12

**Authors:** Jovana M. Jasso-Martínez, Donald L. J. Quicke, Sergey A. Belokobylskij, Bernardo F. Santos, José L. Fernández-Triana, Robert R. Kula, Alejandro Zaldívar-Riverón

**Affiliations:** 1 Colección Nacional de Insectos, Instituto de Biología, Universidad Nacional Autónoma de México, 3er Circuito Exterior s/n, Cd. Universitaria, Copilco, Coyoacán, A. P. 70-233, C. P. 04510 Ciudad de México, México; 2 Posgrado en Ciencias Biológicas, Unidad de Posgrado, Circuito de Posgrados, Universidad Nacional Autónoma de México, Coyoacán, C. P. 04510 Ciudad de México, México; 3 Integrative Ecology Laboratory, Department of Biology, Faculty of Science, Chulalongkorn University, Phayathai Road, Pathumwan, Bangkok, 10330 Thailand; 4 Zoological Institute, Russian Academy of Sciences, St Petersburg, 199034 Russia; 5 Museum and Institute of Zoology Polish Academy of Sciences, 00-679 Warszawa, Poland; 6 Institut de Systématique, Evolution, Biodiversité (ISYEB), Muséum National d’Histoire Naturelle, CNRS, SU, EPHE, UA, 57 rue Cuvier CP50, 75231 Paris Cedex 05, France; 7 Canadian National Collection of Insects, 960 Carling Avenue, Ottawa, ON K1A 0C6 Canada; 8 Systematic Entomology Laboratory, Beltsville Agricultural Research Center, Agricultural Research Service, U.S. Department of Agriculture, C/O Department of Entomology, National Museum of Natural History, Washington, DC USA

**Keywords:** Gene rearrangements, Mitochondrial genome, Phylogenomics, Cyclostome, Non-cyclostome

## Abstract

**Background:**

Mitochondrial (mt) nucleotide sequence data has been by far the most common tool employed to investigate evolutionary relationships. While often considered to be more useful for shallow evolutionary scales, mt genomes have been increasingly shown also to contain valuable phylogenetic information about deep relationships. Further, mt genome organization provides another important source of phylogenetic information and gene reorganizations which are known to be relatively frequent within the insect order Hymenoptera. Here we used a dense taxon sampling comprising 148 mt genomes (132 newly generated) collectively representing members of most of the currently recognised subfamilies of the parasitoid wasp family Braconidae, which is one of the largest radiations of hymenopterans. We employed this data to investigate the evolutionary relationships within the family and to assess the phylogenetic informativeness of previously known and newly discovered mt gene rearrangements.

**Results:**

Most subfamilial relationships and their composition obtained were similar to those recovered in a previous phylogenomic study, such as the restoration of Trachypetinae and the recognition of Apozyginae and Proteropinae as valid braconid subfamilies. We confirmed and detected phylogenetic signal in previously known as well as novel mt gene rearrangements, including mt rearrangements within the cyclostome subfamilies Doryctinae and Rogadinae.

**Conclusions:**

Our results showed that both the mt genome DNA sequence data and gene organization contain valuable phylogenetic signal to elucidate the evolution within Braconidae at different taxonomic levels. This study serves as a basis for further investigation of mt gene rearrangements at different taxonomic scales within the family.

**Supplementary Information:**

The online version contains supplementary material available at 10.1186/s12862-022-01983-1.

## Background

Mitochondria play a central role in cellular metabolism, providing energy to nearly all living eukaryotic organisms [1, 2]. Study of mitochondrial (mt) DNA therefore is fundamental in a number of areas of research, including physiology, molecular biology and evolution [2, 3]. The metazoan mt genome typically consists of 15–18 kilobases, comprising 13 protein-coding genes, 22 transfer RNAs (tRNAs) and two ribosomal RNAs (rRNAs) [4], and this composition is generally conserved across bilaterian metazoans [5, 6].

The analysis of mt nucleotide sequence data is one of the most common approaches to investigate evolutionary relationships. While often considered more useful for shallow evolutionary scales [7], it has been increasingly shown that mtDNA can also be informative to investigate deeper relationships [8, 9]. Another important source of phylogenetic information can be obtained from the mt genome organization, which is generally conserved in many groups of Metazoans but has been shown to be relatively plastic in some insect orders, including Hymenoptera [5, 10, 11].

With more than 21,000 described species [12] distributed in 41 subfamilies [13], Braconidae (Hymenoptera: Ichneumonoidea) represents one of the largest radiations in Hymenoptera [14]. The vast majority of braconid species are either ecto- or endoparasitoids of juvenile stages of other holometabolous insects [14, 15]. Members of this family are divided into two main groups, the cyclostomes sensu lato, which are characterized by having the lower part of the clypeus sharply recessed exposing a concave, smooth glabrous labrum [16], and the non-cyclostomes, which do not have the clypeus sharply recessed and with the labrum flat and setiferous [16]. Cyclostomes *s.l.* include the aphidioid subfamilies and other groups that have secondarily lost the cyclostome condition such as Alysiinae, Opiinae, some Betylobraconini within the subfamily Rogadinae and some Gnamptodontini within Telengaiinae. Cyclostomes *s.l.* and non-cyclostomes together comprise the braconoid complex [13].

The higher-level classification of Braconidae has changed considerably through time, in part because of the challenges posed by high levels of morphological convergence among its members [14], limited taxonomic sampling and/or limited number of available molecular markers (e.g., [11, 17, 18]). For example, some taxa treated as subfamilies within Braconidae (i.e. Apozyginae, Trachypetinae, Masoninae) have been either considered or recovered as separate lineages outside of the family [19–21] but also as part Braconidae based on different sources of evidence [11, 13, 17, 22].

There is a general consensus for several subfamily-level relationships both for the cyclostome and non-cyclostome groups using different sources of data (e.g., [11, 23–26]). In particular, two key molecular phylogenetic studies [24, 26] confirmed a number of subfamilial relationships with strong support, as well as confidently placing a number of genera whose affinities had previously been rather doubtful. However, the placement and relationships of some other genera and subfamilies has remained unclear; for example, relationships of various genera within Hormiinae and Rhysipolinae and also whether some groups (e.g., Doryctinae and Mesostoinae) are actually monophyletic.

Mt gene rearrangements have been used for inferring evolutionary relationships among the braconid subfamilies since the pioneering study of [27], which found a clear pattern of gene rearrangement between the cyclostome and non-cyclostome groups. For the non-cyclostomes, the block of tRNAs located between the protein-coding genes *COX2* and *ATP8* was recovered with a *trnK-trnD* pattern [27]. On the other hand, for the cyclostomes this tRNAs block was recovered with three different arrangements: *trnK-trnD*, *trnD-trnK* and *trnD-trnH-trnK* [27]. Subsequent studies have confirmed the above tRNAs arrangements and phylogenetic relationships among subfamilies that have been recovered in previous studies using protein-coding gene sequence data for phylogenetic reconstruction [25, 28, 29]. Although these studies represent an important basis for investigating the higher-level relationships of Braconidae, their taxonomic sampling was limited, with most of the subfamilies being represented only by one species or not represented at all.

Here, we provide the most comprehensive comparative mitogenomic study of Braconidae carried out to date, using 148 mt genomes (132 newly generated) including species belonging to all cyclostome *s.l.* and most non-cyclostome subfamilies, as well as a number of outgroup taxa. We used protein-coding and rRNA gene sequence data to reconstruct the phylogenetic relationships of Braconidae. We also characterized the organization of the protein-coding, tRNA and rRNA genes at subfamily and tribal levels. We found important variation in the mt gene organization within Braconidae, revealing that this source of data is informative to recognize groups within Braconidae.

## Results

### Mitogenome assembly and annotation

**Fig. 1:**
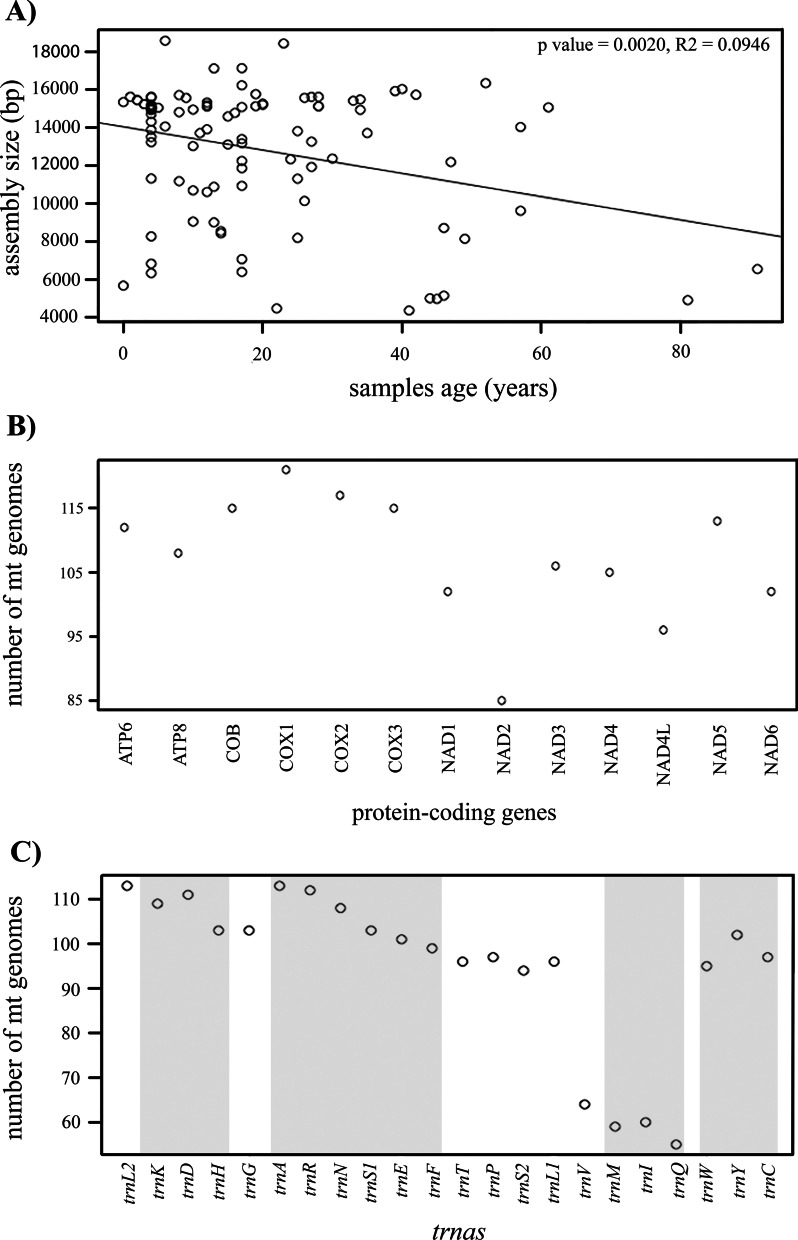
**A** Statistical correlation between the mt genomes assembly size and the age of the examined specimens. **B** Number of mt genomes (y-axis) for which each protein-coding gene was recovered (x-axis). **C** Number of mt genomes (y-axis) for which each tRNA was recovered (x-axis)

We generated and annotated a total of 132 complete and partial ichneumonoid mt genomes (Additional file 1: Table S1). All but 21 mt genomes were recovered in a single contig in the de novo assembly (Additional file 1: Table S1). For cyclostome *s.l.* braconids, we assembled and annotated 29 complete and 58 partial mitogenomes, ranging from 2730 to 19,429 bp with a mean read depth from 8.8 to 870.9. For the non-cyclostome braconids, we generated four complete and 36 partial mitogenomes, ranging from 4364 to 16,033 bp with a mean read depth from 12.1 to 1996.0. For the ichneumonid taxa, we obtained one complete and three partial mitogenomes that ranged from 7924 to 18,583 pb, with a mean read depth from 44.8 to 105.8, whereas we recovered a partial mt genome for *Apozyx penyai* (Apozyginae) that comprised 12,332 bp with a mean read depth = 60.2 (Additional file 1: Table S1). We found a significant correlation between the mt genome assembly size and age of the specimens (p = 0.0020, R^2^ = 0.0946) (Fig. 1A), with specimens between 1–28 and ≥ 30 years having an average of 13,246.45 and 11,174.42 bp assemblies, respectively.

For 66 out of the 122 mt genomes that were assembled as a secondary product of UCE libraries we recovered the two *rRNAs*, for 28 we only recovered the *rrnL*, for three only the *rrnS* and for 25 both rRNAs were missing (Additional file 1: Table S1). The protein coding gene that was recovered for most mt genomes was *COX1* (121 mt genomes), whereas NAD2 was missing for 37 (Fig. 1B, Additional file 1: Table S1). Similarly, for the tRNAs both *trnL2* and *trnA* were recovered for 113 and *trnQ* was recovered for 55 mt genomes (Fig. 1C, Additional file 1: Table S1). Within the tRNA blocks *trnA-trnR-trnN-trnS1-trnE-tnrF*, *trnI-trnM-trnQ*, *trnW-trnY-trnC* and *trnK-trnD-trnH*, the tRNAs *trnA*, *trnI*, *trnY* and *trnD* were recovered in most of the assembled mt genomes, respectively (Fig. 1C).

### Phylogenetic relationships

**Fig. 2:**
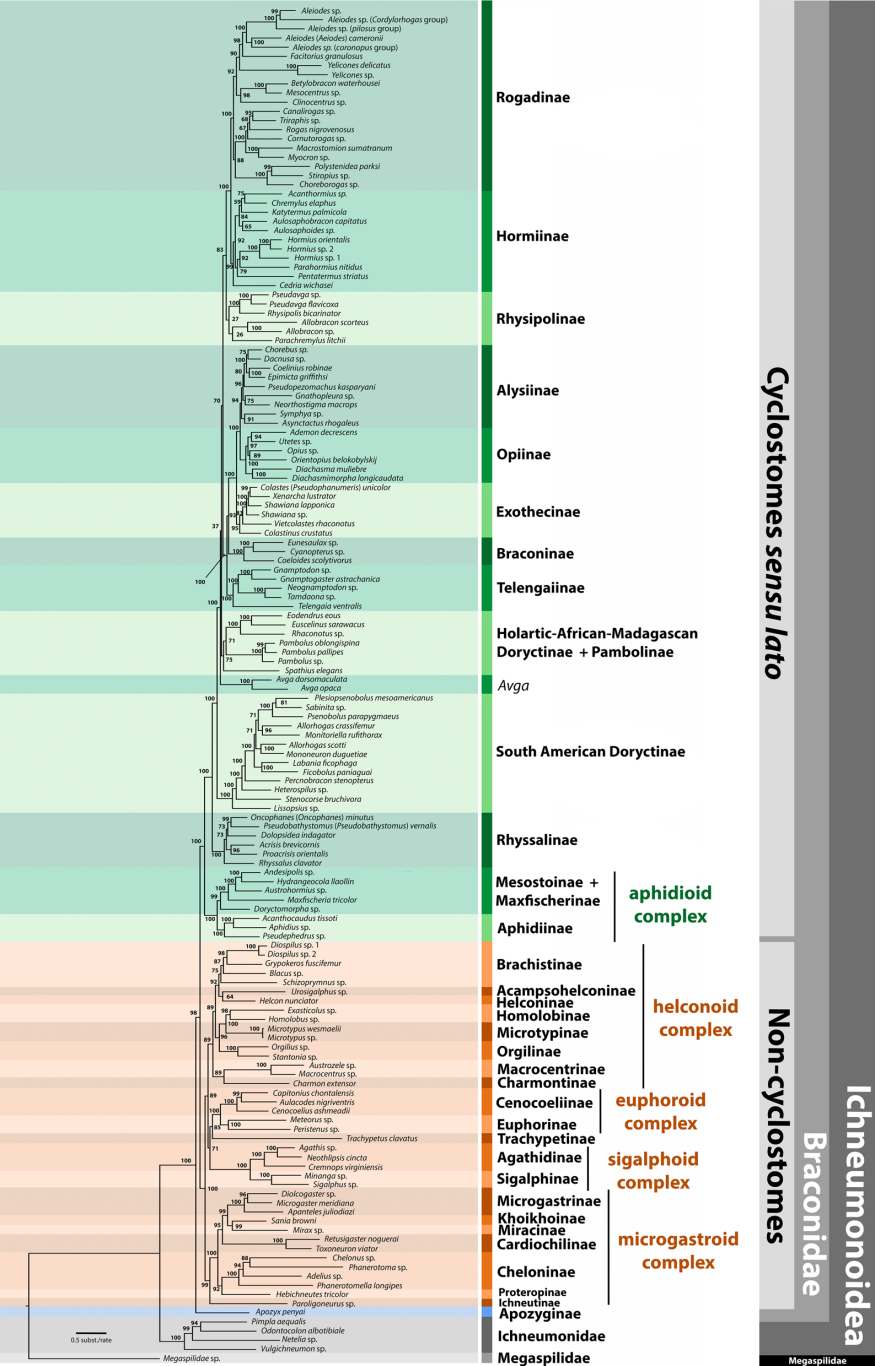
Maximum likelihood phylogram of Braconidae derived from the concatenated matrix with the best-fit partition model. Green = cyclostome *s.l.* subfamilies, orange = non-cyclostomes subfamilies, blue = *Apozyx penyai* (Apozyginae), grey = Ichneumonidae, light grey = Megaspilidae (outgroup). Numbers near nodes are bootstrap values

The phylograms derived from the two ML analyses (predefining partitions and best-fit partitioning scheme) recovered the same topology, with few differences in bootstrap (BTP) values for some nodes (Fig. 2, Additional file 4: Fig. S1). The phylogram derived from the Bayesian analysis recovered almost the same topology obtained in the ML analyses, with most nodes having significantly supported posterior probability (PP) values. Few exceptions were in the placement of the two representatives of *Avga*, which were recovered in the Bayesian phylogram as sister to the clade comprising the Holartic-African-Madagascan (HAM) doryctines + Pambolinae but with low support (PP = 0.52) (Additional file 5: Fig. S2), whereas in the ML topology *Avga* was sister to a large clade including HAM doryctines + Pambolinae but also Rogadinae, Hormiinae, Rhysipolinae and the braconoid subcomplex (Fig. 2, Additional file 5: Fig. S2). Other exceptions were the tribal relationships at the interior of Rogadinae, with some nodes recovered with low support, and the placement of *Spathius elegans* Matthews (Doryctinae) as sister to the braconoid subcomplex, but with low support (PP = 0.40).

The topologies obtained from the analyses with distinct levels of missing data were mostly congruent with the phylograms recovered in the ML and Bayesian analyses using the complete matrix (Additional file 6: Fig. S3). Examples of the same recovered relationships are the monophyly of both cyclostomes and non-cyclostomes, monophyly of most subfamilies (except Doryctinae and Mesostoinae), monophyly of the braconoid subcomplex, sister relationship of the non-cyclostomes with the subfamilies that comprise the aphidioid complex, and the monophyly of the non-cyclostome complexes in the datasets that had taxa representing all four complexes. Finally, in the cases where the only member of Apozyginae was included, it was always recovered as sister to the remaining braconid subfamilies.

Hereafter we refer to the results obtained in the ML analysis conducted using the complete matrix with the best-fit partitioning scheme (Fig. 2), only mentioning the BTP values < 100. Braconidae was recovered as monophyletic and included *T. clavatus* within the non-cyclostome clade. Both cyclostomes and non-cyclostomes were recovered as reciprocally monophyletic. *Apozyx penyai* was recovered as sister to Braconidae, whereas Ichneumonidae was sister to Braconidae + *A. penyai.*

### *Cyclostome s.l. clade*

Most cyclostome subfamilies were recovered as monophyletic with strong support with the exception of Doryctinae and Mesostoinae (with respect to *Maxfischeria tricolor* Papp).

Rogadinae was recovered as sister to Hormiinae. Within Rogadinae, Rogadini was sister to Stiropiini and both were sister to the remaining tribes. Within Hormiinae, Cedriini was strongly supported as sister to the remaining hormiine tribes (BTP = 99). Hormiini and Pentatermini were sister tribes, but Lysitermini was non-monophyletic, with *Aulosaphoides* sister to *Aulosaphobracon capitatus* Belokobylskij and Long (Aulosaphobraconini) but with low support (BTP = 65). Rhysipolinae appeared as sister to Rogadinae + Hormiinae (BTP = 83), and it was divided into two clades, one containing *Pseudavga* and *Rhysipolis bicarinator* Belokobylskij and the other *Allobracon* and *Parachremylus litchii* Belokobylskij and Maeto.

The Alysiinae and Opiinae were sister groups. Within Alysiinae, most representatives of Dacnusini were nested in a single clade except for *Symphya*, which was sister to *Asyntactus rhogaleus* Marshall (Alysiini) (BTP = 91). The opiines *Diachasma muliebre* Muesebeck (Biosterini) and *Diachasmimorpha longicaudata* Ashmead, on the other hand, were recovered as sister taxa, rendering the Opiini as non-monophyletic. Exothecinae was sister to Alysiinae + Opiinae; *Colastes* was recovered as non-monophyletic with *Colastinus crustatus* Belokobylskij as sister to the remaining exothecines. Braconinae was recovered as sister to the Alysiinae + Opiinae + Exothecinae clade with high support (BTP = 93). Telengaiinae was sister to a clade comprising Braconinae, Exothecinae, Opiinae and Alysiinae.

The included members of the Doryctinae were divided into two separate clades. One contained *Eodendrus*, *Euscelinus*, *Rhaconotus* and *Spathius*, together with the members of Pambolinae. The second doryctine clade mainly included Neotropical genera and was sister to all the aforementioned cyclostome braconid subfamilies. The two representative species of *Avga* (Avgini Belokobylskij) were recovered a sister to all the above subfamilies. Rhyssalinae was sister to all aforementioned cyclostome subfamilies, with the acrisidines *Acrisis brevicornis* Hellén and *Proacrisis orientalis* Tobias as sister taxa with strong support (BTP = 96). However, the placement of *Rhyssalus clavator* Haliday as sister to the remaining rhyssalines rendered the tribe Rhyssalini non-monophyletic.

Aphidioid subfamilies were recovered as sister to remaining cyclostomes *s.s*. For instance, *Maxfischeria tricolor* was recovered within Mesostoinae as sister to *Andesipolis* sp., *Hydrangeocola llaollin* Martínez and *Austrohormius* sp. The subfamily Aphidiinae was recovered as sister to Mesostoinae + *M. tricolor*.

#### Non-cyclostome clade

The non-cyclostome clade was recovered as sister to the cyclostome *s.l.* subfamilies. Within the helconoid complex, Brachistinae was sister to Acampsohelconinae + Helconinae (BTP = 92). Homolobinae, Microtypinae and Orgilinae were all nested in a single clade. The Macrocentrinae + Charmontinae clade was recovered as sister to all helconoid subfamilies. Within the euphoroid complex, the relationship Euphorinae + Cenocoellinae was recovered, with *T. clavatus* as its sister taxon with a relatively low support (BTP = 85). The sigalphoid complex, composed of the Agathidinae and Sigalphinae, was sister to the euphoroid complex + *T. clavatus* with low support (BTP = 71).

Two main clades were recovered within the microgastroid complex. One had Cardiochilinae at the base followed by Miracinae + Khoikhoinae + Microgastrinae. In the second clade, Cheloninae was sister to *Hebichneutes tricolor* Sharkey and Wharton (Proteropinae) (BTP = 92), whereas *Paroligoneurus* sp. (Ichneutinae) was sister to the microgastroid subfamilies (BTP = 99).

### Mitochondrial gene patterns

The 13 protein-coding genes were recovered in the same order in the mt genome of all ichneumonoids, with the following two exceptions. In *Chelonus* sp. (Cheloninae), there was an inversion of the *ATP6* and *ATP8* genes, whereas *Stenocorse bruchivora* Crawford (Doryctinae) displayed various translocations (Additional file 2: Table S2).

**Fig. 3:**
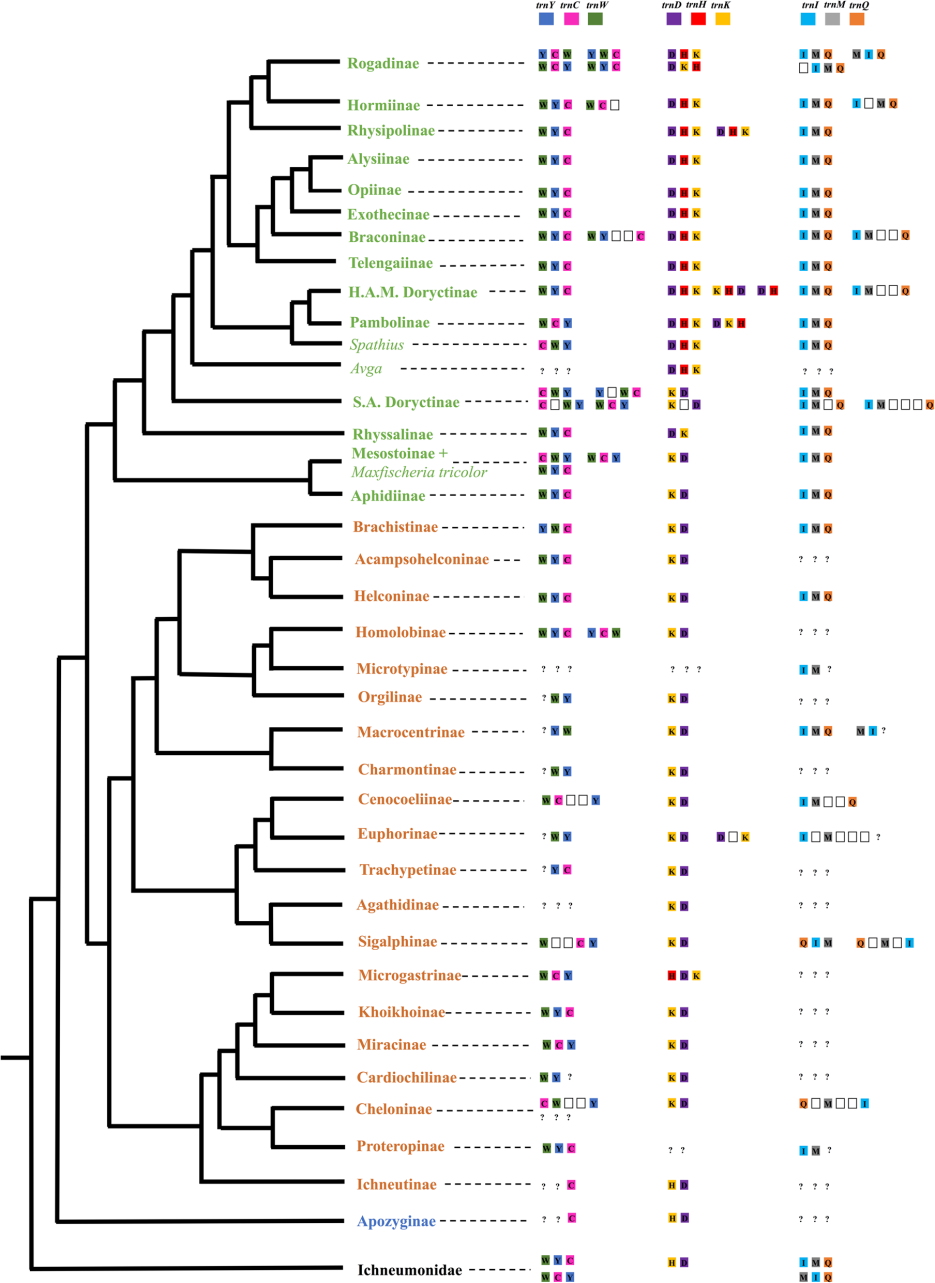
Gene order patterns found for tRNAs clusters *KDH*, *WYC* and *IMQ*. Terminal taxa: green = cyclostome *s.l.* subfamilies, orange = non-cyclostomes subfamilies, blue = *Apozyx penyai* (Apozyginae), black = Ichneumonidae. tRNAs clusters: blue, pink, green = *YCW*, purple, red, yellow = *DHK*, blue, grey, orange = *IMQ*. White squares correspond to other genes (tRNAs or protein coding genes) recovered as part of the *YCW*, *DHK* and *IMQ* clusters. For full results of gene rearrangements, please refer to Additional file 2: Table S2

We found several tRNAs rearrangements. For the tRNAs block surrounding the *NAD2* protein-coding gene we found four different rearrangements for the non-cyclostome subfamilies: (1) *trnW-trnY-trnC,* (2) *trnY-trnC-trnW,* (3) *trnW-trnC-trnY, (4) trnY-trnW-trnC* (Fig. 3, Additional file 2: Table S2). For the cyclostomes *s.l.* (including Aphidiinae, Mesostoinae and Maxfischeriinae), we found six general rearrangements: (1) *trnW-trnY-trnC*, (2) *trnC-trnW-trnY*, (3) *trnW-trnC-trnY*, (4) *trnY-trnC-trnW* and (5) *trnC-trnY-trnW, (6) trnY-trnW-trnC* (Fig. 3, Additional file 2: Table S2).

The tRNA block located between the protein-coding genes *COX2* and *ATP8* showed a *trnK-trnD* pattern for some cyclostome subfamilies (Mesostoinae, Maxfischeriinae, Aphidiinae and neotropical doryctines) and *trnD-trnK* in Rhyssalinae. The *trnK-trnD* pattern was also found for most non-cyclostomes, except for *Meteorus* sp. (Euphorinae) and for the Microgastrinae taxa, which had the *trnD-trnK* and *trnH-trnD-trnK* orders, respectively (Fig. 3, Additional file 2: Table S2). For most remaining cyclostome subfamilies there was a *trnD-trnH-trnK* order, although the orders *trnK-trnH-trnD* and *trnD-trnK-trnH* were also recovered. We found different rearrangements for the tRNA block located between the protein-coding genes *NAD3* and *NAD5*, although for most cyclostome and non-cyclostome subfamilies the *trnA-trnR-trnN-trnS1-trnE-trnF* order was the most common (Additional file 2: Table S2).

We observed tRNAs rearrangement patterns within subfamilies with a better taxon representation. For instance, within Rogadinae we observed rearrangements that were congruent with its tribal classification. The *trnG* was mostly found between the protein-coding genes *COX3* and *NAD3*, although for members of the tribe Rogadini it was found as part of the *trnI, trnM, trnQ* block, located between the rRNAs and the protein-coding gene *NAD2* (Fig. 3, Additional file 2: Table S2). For the clade including Pambolinae + the doryctine genera *Eodendrus*, *Euscelinus*, *Rhaconotus* and *Spathius*, we recovered three different patterns of the tRNAs block between the protein coding genes *COX2* and *ATP8*: (1) *trnD-trnK-trnH*, (2) *trnD-trnH-trnK* and (3) *trnD-trnH* (with the *trnK* located together with *trnI-trnM-trnA-trnQ,* near to the protein coding gene *NAD2*) (Fig. 3, Additional file 2: Table S2). On the other hand, for the Neotropical Doryctinae clade, this tRNA block followed a *trnK-trnD* order, except for *S. bruchivora*, with this block located between the protein coding genes *NAD4L* and *ATP8* including the *trnT* (Fig. 3, Additional file 2: Table S2).

## Discussion

Here we have generated and assembled a large number of mitogenomes for representative species belonging to most subfamilies of Braconidae based on both recently collected and older museum specimens. Our analyses yielded a robustly supported phylogeny that was generally concordant with a recent estimate based on nuclear UCE data [13] thus supporting previous results that mt genome DNA sequence data contain considerable phylogenetic signal at deep-level relationships in insects [8, 9, 30]. Moreover, our comprehensive taxon sampling helped confirm previously known and discover novel gene rearrangements, respectively, which contain phylogenetic signal that correspond with recognized taxa within Braconidae. Below we discuss the most relevant relationships that were supported both by the mitogenome sequence data and gene rearrangements and also highlight the importance that gene reorganization has to unveil the evolutionary history of this megadiverse family.

### Phylogenetic relationships and subfamily level classification in Braconidae

Jasso-Martínez et al. [13] recently proposed 41 braconid subfamilies based on a phylogenomic study with UCE data. Although our study lacked representatives of six subfamilies, the well-supported relationships that were obtained in our best estimate of phylogeny are mostly concordant with those found in the aforementioned study and thus confirmed most of their subfamilial limits and composition.

Our analyses consistently recovered *A. penyai* as sister to the remaining braconid subfamilies. This sister group relationship was also recovered in an ultra-conserved elements (UCE) data study [13]. *Apozyx penyai* possess some morphological features that are absent in nearly all extant braconids but present in Ichneumonidae and some extinct Braconidae [31], such as the presence of fore wing vein 2 m-cu (although also occurring occasionally as an atavism in some Rhyssalinae and Doryctinae) [32–34]. However, it also shares several morphological features with braconids, including the cyclostome condition, fusion of second and third metasomal terga and various venation features [35], thus supporting its placement within the family as the subfamily Apozyginae.

Trachypetidae, consisting of the genera *Trachypetus*, *Megalohelcon* and *Cercobarcon*, was recently elevated to family level based on a phylogenetic study that employed five gene sequence markers and external morphological features [20]. Similar to the Jasso-Martínez et al. [13] study, here we recovered a monophyletic Braconidae with the inclusion of *T. clavatus*. However, we recovered this species as sister to the euphoroid complex without strong support, whereas in the latter nuclear phylogenomic study it was consistently placed as sister to all non-cyclostome subfamilies except Meteorideinae. Trachypetines possess morphological features that are absent in all braconids but are typical of ichneumonids, such as a separation of hind wing veins C and SC + R and the presence of a wing flexion line anterior to hind wing vein M. It also has a small and open fore wing costal cell as in many Cretaceous braconids. Trachypetines also have a well-developed hind wing vein 2-CU typical of ichneumonids but also present in Apozyginae and in the non-cyclostome braconid subfamilies Agathidinae, Sigalphinae, Acampsohelconinae and Meteorideinae [20]. Further studies including members of the two remaining trachypetine genera are necessary to definitively discern the placement of trachypetines within the non-cyclostome clade.

Most of the subfamilial relationships that were strongly supported within Braconidae were concordant with those obtained in other molecular studies [13, 24, 26, 34, 36–38]. Among these are the placement and composition of the aphidioid complex, which we recovered as sister to all other cyclostomes sensu stricto and containing Aphidiinae, Mesostoinae and Maxfischeriinae, with the only member of Maxfischeriinae, *M. tricolor*, deeply nested within Mesostoinae (both cyclostome *s.s*. and aphidioid complex comprising the cyclostome *s.l.* group [13]). Other subfamilial relationships, such as the placement of Rhyssalinae as sister to the remaining cyclostomes *s.s.,* the close relationship and composition of Rhysipolinae, Hormiinae and Rogadinae, and composition of the non-cyclostome subfamily complexes were also confirmed by our mt genome data.

Non-monophyly of the highly diverse subfamily Doryctinae has been recovered both with Sanger sequence and genomic-scale data but with low support [13, 39, 40]. Here we again recovered a non-monophyletic Doryctinae, being divided into two main non-sister clades but with the implied relationships having low support. One of the large main clades included members of the “South American” and the other of the “Holarctic-African-Madagascan” clades that were obtained in Zaldívar-Riverón et al. [40] phylogenetic study of the subfamily, although here the latter clade included the species of the small subfamily Pambolinae. Comprehensive taxon sampling will be needed to elucidate whether Doryctinae as defined traditionally is monophyletic. This subfamily has long been considered hard to diagnose based on derived characters, the row of pegs on the fore tibia often used in subfamily keys being a homoplastic character associated with egress from concealed pupation sites in wood [14]. However, its monophyly is suggested by a small suite of ovipositor tip characters [41] and separate insertions of venom ducts onto the venom reservoir [42].

As has been revealed by numerous previous studies, the Hormiinae sensu lato and the Exothecinae are not closely related, even though they had often been treated as synonymous (e.g., [43]). The genus *Avga* was proposed, together with other genera, to comprise the tribe Avgini, and subsequently it has been placed within Exothecinae, Mesostoinae or Hormiinae [34, 44–49]. Here we recovered *Avga* as sister to a clade comprising Rogadinae, Hormiinae, Rhysipolinae, the braconoid subcomplex and the Holarctic-African-Madagascan (HAM) doryctines + Pambolinae, whereas in the UCE study by Jasso-Martínez et al. [13] it was nested together with *Xenosternum* as sister to the braconoid subcomplex. . Additional studies will thus reveal the phylogenetic affinities of *Avga*, which currently is considered as *incertae sedis* within Braconidae [13].

Based on Sanger sequence data, Sharanowski et al. [26] recovered a clade with intermingled species of Alysiinae, Opiinae, Exothecinae and Telengaiinae (previously Gnamptodontinae), naming it as the alysioid subcomplex. In our analyses, this clade consistently had Braconinae as sister to the former three subfamilies, a relationship that has been recovered in other studies based on analyses of Sanger-sequenced genes [48]. Jasso-Martínez et al. [13] recovered the same taxon composition but with Braconinae as sister to Telengaiinae and renamed it as the braconoid subcomplex.

Four subfamily complexes were considered by Sharanowski et al. [26] within the non-cyclostome lineage—the euphoroid, helconoid, microgastroid and sigalphoid complexes. Our phylogenetic estimates recovered a mainly similar subfamily grouping composition but with different relationships among the complexes in comparison with the Sharanowski et al. [26] and Jasso-Martínez et al. [13] phylogenies. We obtained the microgastroids as sister to the remaining complexes, followed by the helconoids, sigalphoids and the euphoroids + *T. clavatus*. In contrast, in the above two studies the sigalphoids were sister to the microgastroids, and particularly for Jasso-Martínez et al. [13] the only examined member of Meteorideinae was sister to all the non-cyclostomes followed by *T. clavatus* (Trachypetinae), the helconoids and then the euphoroids.

Recently, Jasso-Martínez et al. [13] expanded the composition of the sigalphoid complex to contain Ichneutinae in a restricted sense, including the genera *Ichneutes*, *Oligoneurus* and *Paroligoneurus*, whereas the genera *Hebichneutes*, *Masonbeckia* and *Proterops*, previously placed within Ichneutinae, were included in the subfamily Proteropinae, with the latter being sister to the microgastroid complex. The Proteropinae had also been treated as a subfamily by Chen and van Achterberg [50] and Sharkey et al. [51] based on evidence of previous phylogenetic studies that failed to recover Ichneutinae as monophyletic (e.g., [26]). Nevertheless, Jasso-Martínez et al. [13] phylogenomic study is the only one that has separately recovered both lineages with high support, and therefore, they confirmed Proteropinae as a subfamily. Despite that we had a limited taxon sampling for the non-cyclostome taxa, our results are partially in agreement with the above study, since *Hebichneutes* (Proteropinae) was nested within the microgastroid complex, although *Paroligoneurus* (Ichneutinae) was sister to all microgastroid subfamilies. This contrasts with Jasso-Martínez et al. [13], where Ichneutinae was placed within the sigalphoid group of subfamilies.

### Mt gene rearrangement evolution within Braconidae

Mt gene rearrangements have been shown to be phylogenetically informative at different evolutionary scales in various insect orders, recovering particular patterns for specific lineages [7, 25, 29, 52–55]. In Hymenoptera, sawflies and woodwasps (previously known as Symphyta) usually have a conserved mt gene order, whereas various gene rearrangements have been reported for Apocrita [9, 56].

Previous studies have reported the existence of particular mt gene rearrangements within Braconidae, although taxon sampling in these works was rather limited, only including part of the currently recognised subfamilies and only one or few of their species [11, 25, 27–29]. These studies showed that mt protein-coding gene organization in Braconidae is not substantially different from the putative ancestral Pancrustacean mt genome or among members of this family. Our results confirm this conservative mt protein-coding gene order, as we only found a novel inversion of the *ATP6* and *ATP8* genes in one member of Cheloninae and confirmed previously reported translocations in the doryctine *S. bruchivora* [57]. A conserved protein-coding gene order is also present in other hymenopteran families [7, 56].

In contrast with the protein-coding genes, it has been shown that there are some differences in the tRNAs order pattern between the cyclostome and non-cyclostome subfamilies [11, 25, 27–29]. However, the existence of additional phylogenetically informative tRNAs rearrangements at different levels of divergence was unknown. Our comprehensive taxonomic sampling not only helped confirm the above tRNAs rearrangements but also found that tRNAs reorganizations appear to be consistent with tribes recognized in the two subfamilies with highest species representation, Rogadinae and Doryctinae.

We corroborated the previously observed rearrangements in three main tRNA clusters between members of the cyclostome and non-cyclostome subfamilies, with the non-cyclostomes and the earliest diverging cyclostomes having a more conserved mt gene organization [25, 27, 28]. These tRNAs rearrangements involve the blocks comprising *trnK-trnD-trnH* located between the protein-coding genes *COX2* and *ATP8* [25, 27, 28], the block comprising *trnW-trnC-trnY* near to the protein-coding gene *NAD2* [25] and the block comprising *trnA-trnR-trnN-trnS1-trnE-trnF* located between the protein-coding genes *NAD3* and *NAD5* [58].

We found two novel patterns of tRNA rearrangements that appear to be phylogenetically informative and correspond to tribes within Rogadinae. In one of them, the included species of the tribe Rogadini had a translocation of the *trnG*, which was flanked by the ribosomal *rrnS* locus, as part of the tRNAs cluster *trnI*-*trnM*-*trnQ*. In contrast, the remaining tribes had the putative ancestral condition, where the *trnG* was located between the protein-coding genes *COX3* and *NAD3*. The second rearrangement was detected in the tRNA block situated between the protein coding genes *NAD2* and *COX1*. Within Aleiodini we recovered the *trnY-trnC-trnW* order; we found *trnW-trnC-trnY* for Yeliconini and *trnW-trnY-trnC* for Stiropiini and Betylobraconini. For Rogadini there were three different orders—*trnY-trnW-trnC, trnW-trnC-trnY* and *trnW-trnY-trnC*.

Similar to other phylogenetic studies [13, 40], we recovered the Doryctinae as non-monophyletic, being divided into two separate main clades that were each mainly composed of “South American” (SA) and “Holartic-African-Madagascan” (HAM) genera, respectively. We found two clear differential patterns of tRNAs among these clades between the protein-coding genes *COX2* and *ATP8*. In the HAM doryctine clade, which also included Pambolinae, this tRNAs cluster included *trn*D*, trn*H and *trnK*. On the other hand, similar to the results obtained in Samacá-Sáenz et al. [57], in the SA clade this tRNAs cluster was generally composed of *trnK* and *trnD* except for *S. bruchivora*, whose translocation was located between the protein-coding genes *NAD4L* and *ATP8* with a *trnK-trnT-trnD* order. The *trnK*-*trnD* order observed in the Neotropical doryctine clade was similar to the one found here in the cyclostome *s.l*. subfamilies Rhyssalinae, Aphidiinae, Mesostoinae and Maxfisherinae, as well as in all non-cyclostomes.

### Recovering mt genomes from UCE libraries

The analysis of mt nucleotide sequence data is one of the most common approaches to investigate evolutionary relationships. Generation of mt DNA was until the last decade generally obtained using Sanger sequencing; however, with the advent of next-generation sequencing (NGS), the generation of complete mt genomes has become relatively simple to obtain due the considerably higher efficiency of NGS technologies [30].

In recent years, the sequence capture of UCEs has become one of the most used methods for obtaining genomic-scale data to investigate evolutionary relationships of several animal taxa, including insects (e.g., [59–61]). Regardless of the targeted nature of this technique, raw UCEs datasets can be harvested to recover off-target sequences such as mt DNA, with the possibility of assembled complete mt genomes [62]; thus, the recovery of mt genomes from UCE libraries is currently increasing in phylogenomic studies (e.g., [57, 63]).

In this study, we have shown the efficiency that the raw UCE data have to obtain mt genome sequence data for phylogenomic reconstruction, even when using old and dry museum specimens, since target enrichment methods have shown a higher success rate when working with old museum specimens over other techniques such as RADseq [64]. Here, we recovered shorter assemblies from older samples compared to recently collected samples whose mt genomes were extracted as a secondary product of the UCE data. However, despite the direct relationship between sample age and size of mt assembly, the assembled mitogenomes contained considerable phylogenetic information. As a result we were able to recover a robust estimate of phylogeny, even with a high amount of missing data, that was mostly congruent with a phylogeny obtained using targeted UCE regions (i.e., [13]).

## Conclusions

This comprehensive mt phylogenomic study of Braconidae showed that both the mt genome DNA sequence data and gene organization contain valuable phylogenetic signal that can be employed to elucidate the evolution of this megadiverse group of hymenopterans at different levels of divergence, including deep relationships. This is supported by our phylogenetic reconstruction, which was mostly consistent with previous phylogenetic hypotheses, particularly the one based on nuclear-genome scale data [13]. Moreover, the gene rearrangements discovered in our study can be used as diagnostic features for tribal delimitation within Rogadinae and Doryctinae. Future studies should be carried out with more extensive taxon sampling to discern the existence of phylogenetically informative variation within other braconid subfamilies.

## Methods

### Taxonomic sampling

Our taxon sampling comprised 128 and 143 ingroup genera and species, respectively, covering all biogeographic regions and belonging to most of the extant currently recognized braconid subfamilies (see [13, 14, 37, 50]). We included 102 species from all cyclostome *s.l.* subfamilies and 40 non-cyclostome species comprising the helconoid, euphoroid, sigalphoid and microgastroid complexes (sensu Sharanowski et al., [26]), representing most of the non-cyclostome subfamilies except for Amicrocentrinae, Dirrhopinae, Masoninae, Mendesellinae, Meteorideinae, and Xiphozelinae. We also included a specimen of *Apozyx penyai* Mason. This enigmatic taxon has been placed in its own family, Apozygidae [19] or within Braconidae [13, 17, 31, 65]. We also included a specimen of *Trachypetus clavatus* Guérin-Meneville, which has been placed within Braconidae [66, 67] or elevated as the family Trachypetidae [20], although it was recently returned to Braconidae based on genomic-scale data [13].

We included four species of the family Ichneumonidae as outgroup taxa: *Vulgichneumon* sp. (Ichneumoninae), *Pimpla aequalis* Provancher (Pimplinae), *Netelia* sp. (Tryphoninae) and *Odontocolon albotibiale* Bradley (Xoridinae). We used data from a species of Megaspilidae, of the superfamily Ceraphronoidea, to root the trees. This superfamily was found to be sister to Ichneumonoidea in a recent study based on transcriptomic data [68]. Voucher specimens are housed in the Colección Nacional de Insectos at the Instituto de Biología, Universidad Nacional Autónoma de México (CNIN IB-UNAM); at the Smithsonian Institution National Museum of Natural History, Washington, DC (USNM); in the Zoological Institute, Russian Academy of Sciences, St Petersburg, Russia (ZISP) and at the Canadian National Collection of Insects (CNC), Ottawa, Canada. A list with GenBank accession numbers of the mitogenomes assemblies and further details of all the taxa examined in this study are provided in Additional file 3: Table S3.

### Assembly and annotation of mt genomes

The mt genomes of 122 samples were extracted in silico from raw reads generated from libraries that were originally prepared for obtaining ultra-conserved element (UCE) loci. Details of genomic DNA extraction and library prep protocols are given by Jasso-Martínez et al. [13, 37]. For 10 additional samples (Additional file 1: Tables S1, Additional file 3: Table S3), we used data generated by whole-genome shotgun sequencing. Shotgun libraries were prepared using the Kapa Hyper Prep kit (Kapa Biosystems Inc. Wilmington, MA, USA) and the TruSeq-style dual-indexing adapters [69]. Sequencing was performed in an Illumina HiSeq X Ten instrument at the Department of Environmental Health Science, University of Georgia, Athens, GA, USA.

Raw reads from the UCEs libraries were trimmed and filtered using Illumiprocessor [70], a wrapper around Trimmomatic [71, 72] in the pipeline Phyluce version 1.6.6 [73]. Raw reads from the shotgun sequencing were filtered using Geneious 10.2.6 [74]. Cleaned reads were de novo assembled into the mt genome sequence with the GetOrganelle toolkit [75] using the default database ‘animal_mt’. For the datasets from which we did not recover the complete mitogenome or obtained more than one contig in the de novo assembly, we used a combination of assembly approaches in order to obtain longer contigs as follows. For a given sample, the contig(s) obtained in GetOrganelle were used as template to obtain a unique and longest contig using by-reference assembly in the program Geneious 10.2.6 [74]. We avoided using as template the assembled mitogenome from a different sample, even if closely related, so as to not bias the specific gene order of each individual.

The mt sequences of 14 doryctines and *Pambolus oblongispina* (Pambolinae) that were generated in Samacá-Sáenz et al. [57] study (Additional file 3: Table S3) were downloaded from GenBank and annotated together with the assemblies obtained in this study in the MITOS 2 webserver [76] using the invertebrate genetic code. We verified the protein-coding genes signal from the “protein plots” generated by MITOS. Finally, we used the program Geneious version 10.2.6 [74] to confirm the accuracy of our assemblies and annotations. We registered the order of the protein-coding genes, tRNAs and rRNAs to identify patterns of gene rearrangements using as reference the Pancrustacea ground pattern, which is the proposed Crustacea/Hexapoda common ancestor [77, 78].

Several of the museum specimens employed in this study were of considerable age. We therefore investigated the correlation between specimen’s age (0—91 years old) with the mt genomes assembly size calculating the Pearson’s correlation coefficient of these variables with R version 3.6.0 [79]. We also used R to plot the number of mt genomes for which each protein-coding and *tRNA* genes were recovered. For both the statistical tests and plots, we excluded the mt genomes of samples that did not have a collection date, that we did not assemble in this study (i.e., most doryctines and *Pambolus oblongispina* [57]), as well as those that were assembled from shotgun libraries.

### Matrix alignment and phylogenetic analyses

We extracted for all samples the 13 protein-coding and the two ribosomal RNAs (rRNAs) sequences. The alignments of the protein-coding genes were performed independently (13 alignments) with the program MAFFT version 7 [80]. We verified the protein-coding gene alignments with respect to the reading frame (invertebrate mt genetic code). Some regions of the translated alignments had unalignable regions. These ambiguities were delimited by identifying the conserved flanking regions and removed. The mt rRNA gene regions were aligned according to Wu et al. [81] model with additional reference to Buckley et al. [82]. The 16S gene was aligned between the core I region and five bases after H2675, a length comprising approximately 1140 bases of which 763 were considered reliably alignable. For 12S, we considered approximately 620 bases between H500 until 7 bases following the H1506 helix. Of these, 340 bases were reliably alignable. In both cases, the analyzed reliably alignable positions included a mix of base-pairing helix stems, as well as length conserved loops, expansion regions and stretches of core sequence. The alignable bases of the ribosomal genes and the 13 protein-coding gene alignments, a total of 15 genes for the complete matrix, were concatenated in the program Geneious version 10.2.6 [74].

We predefined 41 partitions for the concatenated matrix: three partitions based on codon position for each of the 13 protein-coding genes and one partition each for the two rRNA genes. We selected the best-fit partitioning scheme and substitution model with ModelFinder [83] in the program IQTREE version 2 [84] according to the Bayesian information criterion, obtaining 17 subsets of partitions. We conducted two Maximum Likelihood (ML) analyses in IQTREE version 2 [84] with 1000 ultra-fast bootstrap replicates using (1) the matrix with the 41 predefined partitions based on codon position and rRNAs, and (2) the matrix with the best-fit partitioning scheme. The concatenated alignment consisted of 148 terminal taxa and 11,717 base pairs. For the matrix with the best-fit partitioned scheme we also conducted a Bayesian analysis with the program Mr. Bayes version 3.2.7 [85], which consisted of two simultaneous runs of 50 million generation each, sampling trees every 5000 generations and a burn-in fraction of 0.25. The concatenated alignment including partition sets and the annotated alignments of the used ribosomal genes 16S and 12S are available as Additional file 7: File S1, Additional file 8: File S2 and Additional file 9: File S3, respectively.

We evaluated whether different levels of missing data and number of taxa had an effect on our phylogenetic inferences. For this, we generated four additional datasets considering the number of missing genes as follows: (1) dataset including taxa with no missing genes, (2) dataset including taxa with 0–2 missing genes, (3) dataset including taxa with 0–6 missing genes and (4) dataset including taxa with 0–9 missing genes. Therefore, for each dataset, we included 70, 105, 128 and 142 taxa including the outgroup, respectively. For each dataset we selected the best-fit partitioning scheme and substitution model with ModelFinder [83] and performed ML analyses in IQTREE version 2 [84] with 1000 ultra-fast bootstrap replicates. All four matrices and their included partition sets are available in a single file as Additional file 10: File S4.

## Supplementary Information

**Additional file 1: Table S1.** Main features of the assembled mitochondrial genomes in this study: assembly sizes (pb), mean read depth, number of contigs obtained in GetOrganelle (GO) and not-found genes. Samples with an asterisk (*) correspond to the samples from which the mt assembly was obtained from shotgun libraries.

**Additional file 2: Table S2.** Gene order of the mt genomes assembled in this study. The protein-coding genes, tRNAs and rRNAs are highlighted in blue, white and purple respectively. Grey cells correspond to not-recovered genes. Samples with asterisks (*) were sequenced and assembled by Samacá-Sáenz et al. (2019).

**Additional file 3: Table S3.** List of specimens included in this study. Their taxon ID, locality, biogeographic region, raw UCEs data and mitogenomes GenBank accession numbers. SRA accession numbers marked with asterisks (*) correspond to shotgun data.

**Additional file 4: Figure S1.** Maximum likelihood phylogram of Braconidae derived from the concatenated matrix with the predefined partitions. Green = cyclostome *s.l.* subfamilies, orange = non-cyclostomes subfamilies, blue = *Apozyx penyai* (Apozyginae), grey = non-braconids. Numbers near nodes are bootstrap values.

**Additional file 5: Figure S2.** Bayesian phylogram of Braconidae derived from the concatenated matrix with the predefined partitions. Green = cyclostome *s.l.* subfamilies, orange = non-cyclostomes subfamilies, blue = *Apozyx penyai* (Apozyginae), grey = non-braconids. Numbers near nodes are posterior probability (PP) values ≤ 0.95; nodes with no value have PP ≥ 0.95.

**Additional file 5: Figure S3.** Maximum likelihood phylograms of Braconidae derived from four dataset to test the level of missing data. **A** 0–9 missing genes, 142 taxa, **B** 0–6 missing genes, 128 taxa, **C** 0–2 missing genes, 105 taxa and **D** no missing genes, 70 taxa.

**Additional file 7: File S1.** Concatenated matrix including the sequences of the 13 protein-coding genes + the sequences of the two ribosomal RNAs (11,717 pb, 148 terminals), as well as the block of the estimated partitions.

**Additional file 8: File S2**. Annotated 16S matrix.

**Additional file 9: File S3.** Annotated 12S matrix.

**Additional file 10:** **File S4.** Datasets used for missing data evaluation.

## Data Availability

All examined datasets are available as supplementary materials, raw data is available for download in the Sequence Read Archive of the National Center for Biotechnology Information (SRA-NCBI) under the BioProject PRJNA795146. The mt genome sequences are available in NCBI; accession numbers are provided in Additional file3: Table S3.

## Abbreviations

mt: Mitochondrial
mtDNA: Mitochondrial DNA
mt genome(s): Mitochondrial genome(s)
tRNAs: Transfer RNAs
rRNAs: Ribosomal RNAs
*trn*: A particular tRNA
*ATP6*: Mitochondrial synthase membrane subunit 6
*ATP8*: Mitochondrial synthase membrane subunit 8
*COX1*: Cytochrome c oxidase subunit I
*COX2*: Cytochrome c oxidase subunit II
*COX3*: Cytochrome c oxidase subunit III
*NAD2*: NADH dehydrogenase 2
*NAD3*: NADH dehydrogenase 3
*NAD4*: NADH-ubiquinone oxidoreductase chain 4
*NAD4L*: NADH-ubiquinone oxidoreductase chain 4L
*NAD5*: NADH-ubiquinone oxidoreductase chain 5
*rrnS*: Mitochondrial small subunit ribosomal RNA (12S)
*rrnL*: Mitochondrial large subunit ribosomal RNA (16S)
*s.s*.: Sensu stricto
*s.l*.: Sensu lato
bp: Base pairs
BTP: Bootstrap
UCE(s): Ultra-conserved element(s)
SA: South American
HAM: Holartic-African-Madagascan
NGS: Next-generation sequencing
